# A qualitative study of facilitators and barriers to intraoperative acquired pressure injury prevention implementation in operating room nurses based on the CFIR framework

**DOI:** 10.3389/frhs.2026.1877556

**Published:** 2026-08-24

**Authors:** Yuan Gao, Juan Chen, Wenhui Guang, Wenli Xu, Zhibin Wei, Hongying Yang

**Affiliations:** 1 Clinical Medical College, Qinghai University, Xining, Qinghai, China; 2 Clean Operating Room, Qinghai Provincial People’s Hospital, Xining, Qinghai, China; 3 Anesthesiology, Qinghai Provincial People’s Hospital, Xining, Qinghai, China

**Keywords:** operating room nurses, intraoperative acquired pressure injury, consolidated framework for implementation research, facilitators, barriers, qualitative study

## Abstract

**Objective:**

Intraoperative acquired pressure injury (IAPI) is a common perioperative complication in surgical patients, and operating room nurses are the primary implementers of evidence-based IAPI prevention strategies. Although standardized evidence-based guidelines for IAPI prevention exist, their translation into clinical practice remains suboptimal. Guided by the Consolidated Framework for Implementation Research (CFIR), this study aimed to identify barriers and facilitators to implementing these prevention measures, to provide an empirical basis for the clinical translation of evidence-based IAPI prevention interventions.

**Methods:**

A descriptive qualitative research design was adopted. A semi-structured interview guide was developed in strict alignment with the CFIR 2.0 framework. Twelve operating room nurses from a tertiary general hospital in Qinghai Province were recruited via purposive sampling, and face-to-face individual interviews were conducted for data collection. Qualitative content analysis was used to code the data, and core themes were extracted from the full dataset.

**Results:**

With reference to the five core domains of the CFIR framework, 14 key factors influencing the implementation of evidence-based IAPI prevention measures were identified, categorized into 6 barriers and 8 facilitators. The barriers included: mismatch between evidence-based guidelines and operating room clinical realities; insufficient protective supplies; lack of targeted positive incentives; poor interdepartmental communication; uneven prevention competencies among nurses; and experience-dependent practice with lax adherence among some nurses. The facilitators included: multi-channel access to evidence-based information; industry policy support; aligned prevention goals among nurses; accessible professional training and guidance; emphasis and support from department managers; clinical adaptation needs; flexible implementation strategies; and nurses’ awareness of self-reflection and improvement.

**Conclusion:**

The clinical implementation of evidence-based IAPI prevention measures in operating rooms is influenced by multilevel factors. The core challenges lie in the mismatch between general evidence-based guidelines and the specialized operating room setting, as well as the absence of positive incentive mechanisms. This study fills a research gap in this field and provides practical evidence for establishing a specialized evidence-based practice system for IAPI prevention.

## 1 Introduction

Intraoperative acquired pressure injury (IAPI) refers to pressure injuries (PI) that occur during surgery in the skin and subcutaneous tissue at sites of pressure, typically located at bony prominences or at interfaces where medical devices or equipment come into contact with the patient (1). Surgical patients are at high risk for developing pressure injuries (2). Global studies report that the incidence of IAPI in operating rooms ranges from 5.8% to 66.0%, depending on the type of surgery and the patient's condition (1, 3). The occurrence of IAPI can cause negative emotional distress and significant pain for patients, as well as prolong their hospital stay (4). As IAPI progresses, the incidence of postoperative complications also increases. Studies (5) indicate that patients with IAPI exhibit an upward trend in readmission rates and mortality within 30 days post-surgery. Compared to general clinical wards, preventing IAPI in surgical patients is particularly challenging due to specific surgical factors such as the need for prolonged immobilization during surgery, fluctuations in physiological status, and anesthesia (6, 7). The operating room is both the primary setting for IAPI occurrence and a critical stage for implementing preventive measures. Consequently, IAPI is given high priority in the nursing profession and is regarded as one of the key indicators for evaluating the quality of surgical nursing care.

Given the high incidence and high treatment costs of IAPI, numerous guidelines and consensus statements have been published worldwide specifically aimed at preventing IAPI. Leading international organizations (8, 9) have developed and updated evidence-based guidelines and systematic strategies for the prevention and control of IAPI, emphasizing a comprehensive, full-cycle management model that encompasses assessment, prevention, treatment, and rehabilitation. The American Society of Perioperative Nurses and the Chinese Nursing Association (10, 11) have pointed out that interdisciplinary teams and continuous training have a significant impact on the effectiveness of IAPI prevention. Although clear guidelines and well-established prevention methods are currently available, issues such as inadequate implementation of preventive measures and inconsistent execution standards remain widespread in actual clinical surgical practice (12). As the direct implementers of patient prevention measures, operating room nurses' understanding and adherence to intraoperative prevention protocols directly influence patient clinical outcomes. However, operating room nurses exhibit deficiencies in their knowledge, attitudes, and behaviors regarding IAPI prevention. Studies (13, 14) indicate that operating room nurses generally lack adequate awareness of IAPI prevention. Furthermore, there is a disparity between nurses' positive attitudes toward IAPI prevention and their actual actions (13, 15). Current research on IAPI prevention has largely focused on optimizing prevention strategies, neglecting the critical role of operating room nurses in the implementation process (5, 16). Although one study (17) noted that enhancing nursing staff's preventive awareness and optimizing operating room care models can improve the implementation of standardized care. However, no study has yet employed a rigorous theoretical framework to systematically investigate the barriers and facilitators that operating room nurses face when implementing IAPI prevention measures in their daily work. The Consolidated Framework for Implementation Research (CFIR) is one of the most widely used theoretical frameworks in the field of implementation science, designed to assess and identify the barriers and facilitators that influence the effectiveness of evidence-based practice implementation (18). CFIR categorizes implementation-related factors into five domains: innovation characteristics, external factors, internal factors, individual characteristics, and the implementation process. CFIR has been widely applied in clinical implementation studies across healthcare institutions at all levels, successfully identifying barriers and facilitators for the implementation of multiple evidence-based practices, including emergency department screening, ERAS protocols, perioperative hypothermia prevention, and ICU weaning strategies (19–21). A literature review revealed that no studies have yet used the CFIR framework to systematically analyze the key factors influencing operating room nurses' implementation of IAPI prevention measures. Therefore, guided by the CFIR 2.0 framework and employing a descriptive qualitative research method, this study conducted semi-structured interviews to thoroughly explore the facilitators and barriers to operating room nurses' implementation of evidence-based IAPI prevention measures, aiming to provide a reference for the clinical translation of evidence-based IAPI prevention and control measures.

## 2 Materials and methods

### 2.1 Study design

This study adopted a qualitative descriptive design. One-to-one, face-to-face semi-structured interviews were conducted to explore barriers and facilitators influencing changes in clinical nursing practice (22). Reporting of the study followed the Consolidated Criteria for Reporting Qualitative Research (COREQ) checklist, which was submitted as a supplementary file. The Consolidated Framework for Implementation Research (CFIR 2.0) was used as the theoretical framework. The interview guide was developed based on the five core CFIR domains to ensure a comprehensive and systematic exploration of implementation determinants.

The development of the interview guide followed a standardized procedure (23). First, a systematic literature review was conducted and integrated with clinical practice experience and the study objectives. An initial draft of the interview guide was developed through multiple rounds of discussion within the research team. Subsequently, pilot interviews were conducted with two nurses who met the inclusion criteria. Based on feedback from the pilot interviews, the wording and structure of the questions were refined for clarity and relevance. Finally, the interview guide was reviewed for content validity by two independent experts, including one operating room nursing specialist and one experienced qualitative researcher. The guide was finalized after incorporating their feedback (Table 1).

**Table 1:** Formal interview outline.

NO.	Interview Questions
1	What are your primary sources of evidence-based information regarding the prevention of intraoperative pressure injury?
2	How well do you find these evidence-based preventive measures align with your actual clinical practice? In your daily work, what specific preventive measures do you implement to prevent intraoperative pressure injury? What is the rationale for selecting these measures?
3	Have any quality control standards, management requirements, or performance metrics related to intraoperative pressure injury prevention been established at the hospital level or within the nursing profession?
4	What management policies does your department currently have in place regarding intraoperative pressure injuries? How effective is the implementation of these policies?
5	To what extent do your department and management prioritize the prevention of intraoperative pressure injury?
6	When implementing intraoperative pressure injury prevention measures, what factors do you believe help you carry out this work more effectively?
7	When implementing intraoperative pressure injury prevention measures, what factors limit your ability to carry out these preventive efforts?
8	How do you view the notion that intraoperative pressure injuries are “inevitable”? Does this perception affect your enthusiasm and initiative in carrying out prevention efforts?
9	What emotions do you experience when a patient under your care develops an intraoperative pressure injury? Do you believe specialized training on intraoperative pressure injuries is necessary? If so, what topics would you like the training to cover?
10	When implementing intraoperative pressure injury prevention measures, what kind of support would you like your department or hospital to provide?
11	Regarding the comprehensive perioperative management of intraoperative pressure injury (preoperative assessment, intraoperative prevention, postoperative handover, and follow-up), what issues do you identify with the current implementation process? What specific recommendations do you have to address these issues?

### 2.2 Theoretical framework used: CFIR

The Comprehensive Framework for Implementation Research was developed by Damschroder et al. (18). In 2022, the framework's dimensions were refined based on global user feedback and practical validation, ultimately resulting in CFIR 2.0. CFIR 2.0 comprises five core domains, 48 core constructs, and 19 sub-constructs, designed to adapt to complex, multi-level implementation scenarios. This framework has been widely applied across various healthcare fields. In the field of specialized nursing, it has been used for the clinical translation of evidence-based nursing practices, such as perioperative safety management in operating rooms and self-management for patients with diabetes (24, 25). In the public health sector, it encompasses the implementation and evaluation of public health interventions such as COVID-19 prevention and control, telemedicine services, and chronic disease screening (26, 27). In the field of multidisciplinary collaboration, it covers the optimization of interdisciplinary service models such as multidisciplinary tumor diagnosis and treatment (MDT) and comprehensive geriatric assessment (28, 29).

### 2.3 Research background and participants

This study employed purposive sampling (30) to select 12 nurses currently working in the clean operating room of a tertiary general hospital in Qinghai Province, China, as study participants. This clean operating room is a leading perioperative care center in the Qinghai region, equipped with 30 standard clean operating rooms and performing over 20,000 surgeries annually. It covers all types of high-risk surgeries for IAPI, and the department's nurses possess extensive frontline clinical experience in IAPI prevention, which aligns closely with the research objectives of this study. The inclusion criteria for this study were: holding a valid nursing license issued by the People's Republic of China; working full-time in the operating room with at least 3 years of operating room nursing experience; and being a nursing professional directly involved in perioperative pressure injury prevention care who voluntarily agreed to participate in this study. Among the nurses meeting the inclusion criteria, 3 declined to participate due to reluctance to be recorded, and 1 declined due to a busy clinical schedule that prevented coordination with the interview time. Ultimately, 12 subjects were included in the study. This study was approved by the Ethics Committee of the investigators' hospital (Ethics Approval No.: 2025-377-04) and was conducted in strict accordance with the Declaration of Helsinki. All participants were fully informed of the study purpose, procedures, risks and benefits prior to enrollment, and all provided written informed consent voluntarily. Participants were assured of their right to withdraw from the study at any time without negative consequences.

The first author, who conducted all face-to-face interviews, was a nursing researcher affiliated with the same hospital but had no direct line-management authority over the operating room nursing team. No hierarchical supervisory relationship existed between the interviewer and the participants.

To mitigate the risk of social desirability bias, participants were explicitly assured of complete anonymity and confidentiality of their responses before the interview, and were informed that their answers would not be shared with department managers or affect their performance evaluation. The interviewer maintained a neutral, non-judgmental stance throughout the interviews and used open-ended questions to encourage candid expression of views.

### 2.4 Data collection

Semi-structured interviews were conducted between January and February 2026. All interviews were carried out in a quiet, dedicated meeting room within the operating room department to minimize environmental distractions and ensure participant comfort. Approximately 24–48 h prior to each interview, the first author contacted participants either in person or via telephone/online communication. The study purpose, procedures, interview format, and audio-recording procedures were fully explained. Written informed consent was obtained from all participants prior to participation, and interview appointments were scheduled accordingly. Given participants' clinical workload and daily working conditions, each interview lasted approximately 15–30 min. During the interviews, participants were encouraged to freely express their experiences and perceptions. Probing questions were used when necessary to clarify or deepen responses. Non-verbal information, including tone, emotional expressions, and behavioral cues, was also recorded to enrich contextual understanding of the data. At the beginning of each interview, demographic and professional characteristics were collected, including age, gender, educational level, professional title, nursing hierarchy, current clinical area, and years of operating room experience. These data were used to describe participant characteristics and support contextual analysis of findings.

Data saturation was assessed using the Comparative Method for Themes Saturation (CoMeTS) proposed by Constantinou et al. (31), with the pre-defined criterion that saturation was achieved when no new codes, themes or CFIR constructs emerged across two consecutive interviews. In this study, data collection and analysis were conducted concurrently. By the 10th interview, all core constructs related to barriers and facilitators of IAPI prevention had been identified, and no new themes or conceptual constructs emerged. Two additional interviews (participants 11 and 12) were conducted to confirm thematic stability, which only provided supplementary details and validation of existing themes. Therefore, thematic saturation was confirmed with a sample size of 12, which was sufficient to address the research objectives.

### 2.5 Data analysis

Within 24 h of each interview, research team members transcribed the interview data verbatim using iFly Simultaneous Interpretation software. The transcribed texts were immediately returned to the corresponding interviewees for content verification to ensure alignment with the interviewees' original intent. NVivo 12 software was adopted for data management, and all participant information was anonymized and labeled as P1–P12. Data analysis employed an iterative qualitative content analysis method combining deductive and inductive approaches, with the entire process strictly adhering to the qualitative research analysis standards proposed by Elo et al. (32). During the deductive analysis phase, the research team first established a predefined analysis matrix based on the five core domains of CFIR 2.0. Constructs were mapped to CFIR 2.0 domains according to semantic correspondence and conceptual fit; specifically, a construct was considered valid and retained in the final analytical framework only when it was explicitly mentioned by at least two participants and had a clear impact on the implementation of IAPI prevention measures, while constructs unmentioned by participants or with no perceived influence on implementation were excluded. Subsequently, the full interview transcripts were repeatedly reviewed to identify text units of research significance, which were then mapped to the corresponding predefined dimensions to ensure the entire analysis process remained closely aligned with the study's theoretical framework. During the inductive analysis phase, all valid text units first underwent initial open coding, followed by the consolidation and categorization of semantically similar codes. After repeated comparison and mapping to the most appropriate CFIR 2.0 core domains, core themes capable of addressing the research questions were ultimately identified through in-depth interpretation and theoretical refinement of these categories.

### 2.6 Feasibility

To ensure the rigor and credibility of the qualitative analysis, we employed multiple standardized strategies. First, member checking was conducted to verify the authenticity of the data. Within 24 h after each interview, all transcripts were returned to the corresponding participants for content review to confirm that the transcriptions accurately reflected their original perspectives. Second, coding reliability was strictly controlled. Two trained researchers (GY, XWL) independently coded all eligible transcripts according to a predefined CFIR 2.0 analysis matrix. Inter-coder agreement was calculated using Cohen's kappa coefficient, with an initial kappa value of 0.82, indicating substantial agreement. Discrepancies in coding were first resolved through discussion between the two coders. If consensus could not be reached, a third senior researcher (WZB) with extensive qualitative research experience was consulted for a final decision. After consensus reconciliation, the final coding framework was uniformly applied across all transcripts.

## 3 Results

### 3.1 Participant characteristics

This study included 12 operating room nurses as interview participants. The age range of the participants was 30–48 years, with a mean age of (35.92 ± 4.36) years; 10 were female (83.33%) and 2 were male (16.67%); all participants held a bachelor's degree; the majority held the position of Senior Nurse (9, 75%), while 3 were Registered Nurses (25%); In terms of nursing levels, most were N3 (6, 50%), with 3 each at N1 and N4 (25% each); their practice areas covered nursing care for surgeries across multiple specialties, including general surgery, head and neck surgery, cardiothoracic surgery, orthopedics, and obstetrics and gynecology; years of experience ranged from 7 to 30 years, with a mean of (14.75 ± 5.68) years see Table 2 for details.

**Table 2:** Respondent demographic survey.

No.	Age	Gender	Education	Job title	Nurse level	Current work area	Years of work experience
1	48	Female	Bachelor's degree	Nurse-in-Charge	N3	General surgery	30
2	36	male	Bachelor's degree	Nurse-in-Charge	N3	Urology, Liver transplantation	14
3	38	Female	Bachelor's degree	Nurse-in-Charge	N4	Head and neck surgery	16
4	33	Female	Bachelor's degree	Registered Nurse	N3	General surgery	13
5	30	male	Bachelor's degree	Registered Nurse	N1	Cardiothoracic surgery	8
6	36	Female	Bachelor's degree	Nurse-in-Charge	N4	Cardiothoracic interventional surgery	14
7	39	Female	Bachelor's degree	Nurse-in-Charge	N4	Orthopedic surgery	16
8	36	Female	Bachelor's degree	Nurse-in-Charge	N3	General surgery, Liver transplantation	15
9	32	Female	Bachelor's degree	Registered Nurse	N1	Obstetrics and Gynecology surgery	7
10	36	Female	Bachelor's degree	Nurse-in-Charge	N1	Obstetrics and Gynecology surgery	15
11	36	Female	Bachelor's degree	Nurse-in-Charge	N3	General surgery	14
12	37	Female	Bachelor's degree	Nurse-in-Charge	N3	Head and neck surgery	15

### 3.2 Qualitative research findings

**Figure 1:**
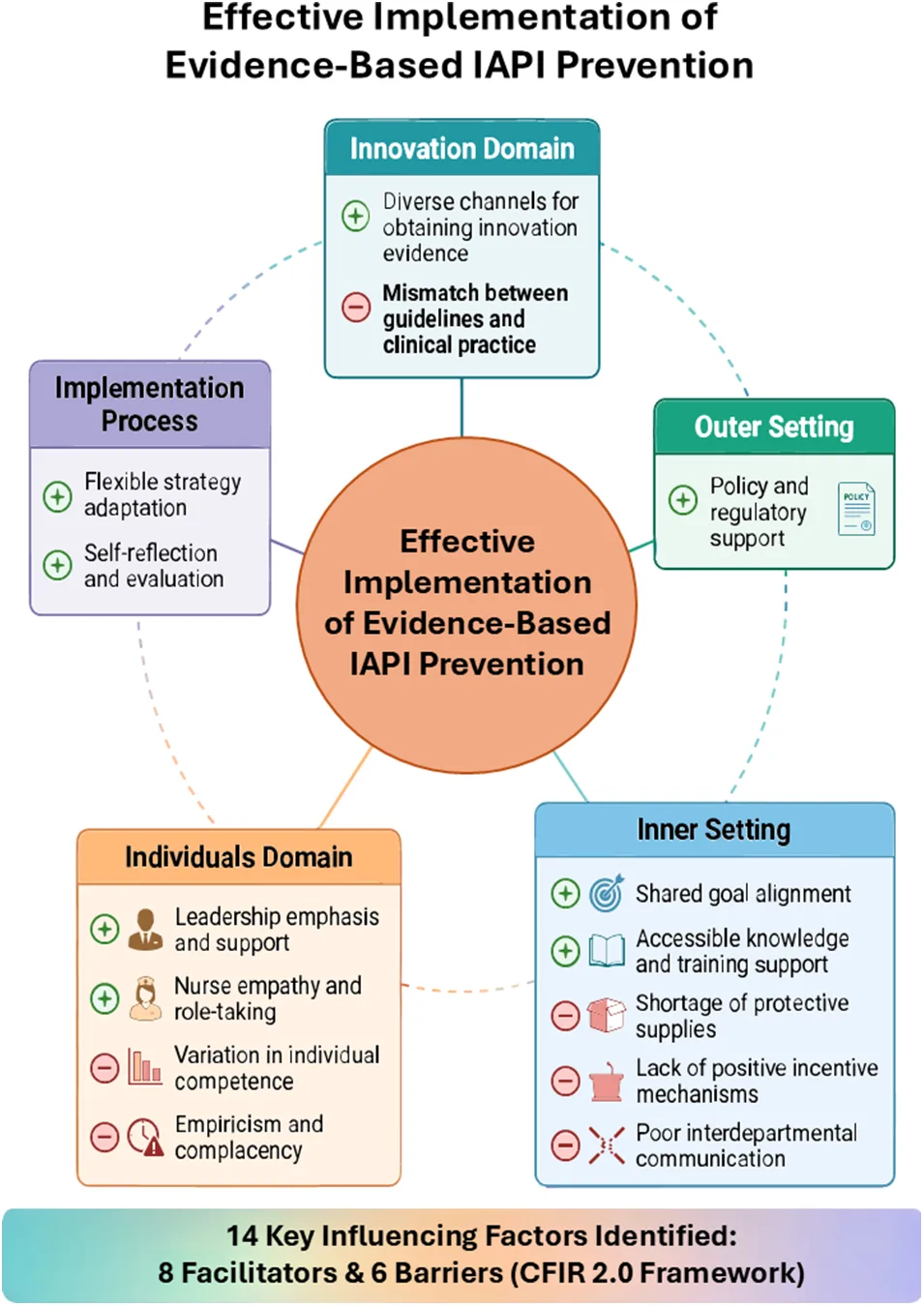
Identified influencing factors for intraoperative acquired pressure injury prevention among operating room nurses mapped to the CFIR 2.0 framework.

From the 48 CFIR constructs and 19 sub-constructs, this study identified 14 constructs across five domains that the research team found to be closely related to the implementation of IAPI prevention measures in the hospitals surveyed see Figure 1. Among the 14 constructs, six barriers and eight facilitators were identified see Tables 3, 4 for details.

**Table 3:** Barriers to the prevention of intraoperative pressure injuries by operating room nurses.

CFIR domains and structures	Barriers
I. Innovation domain	
Innovation applicability	Discrepancy between theory and clinical practice
II. Internal environment	
Available resources	Shortages of departmental supplies
Incentive mechanisms	Lack of positive incentive mechanisms
Communication	Poor cross-departmental communication channels
III. Individual characteristics	
Capabilities	Variations in individual capabilities
Motivation	Presence of empiricism and complacency

Domain II (Outer Setting) and Domain V (Implementation Process) are not presented in this table, as no barriers were identified in these two domains.

**Table 4:** Factors contributing to the prevention of intraoperative pressure injuries by operating room nurses.

CFIR domains and framework	Facilitating factors
I. Innovation domain	
Sources of innovation	Diversification of channels for obtaining evidence of innovation
II. External environment	
Policies and laws	Strong policy and regulatory support
III. Internal environment	
Alignment of goals	Shared expectations to reduce the occurrence of IAPI
Accessibility of knowledge and information	Availability of guidance or training to implement and promote innovation
IV. Individual characteristics	
Leaders	Leadership support
Needs	Empathy for the patient experience
V. Implementation	
Strategy adjustment	Adjusting implementation strategies based on actual circumstances
Reflection and evaluation	Engaging in effective self-reflection

#### 3.2.1 Theme 1: innovation domain

##### 3.2.1.1 Sources of innovation (+): diversification of channels for obtaining innovation evidence

Innovation evidence is key to ensuring that innovations are actually implemented and put to use. All respondents in this study actively seek out innovation evidence related to IAPI through multiple channels. P6: “The first source is hospital training, which is where I gain most of my knowledge; the second is what I learned during my academic studies. Additionally, when I encounter issues at work, I conduct targeted learning by searching for relevant materials online; the hospital also provides learning resources in this area—those are the main sources.” P8: “I have obtained such evidence, primarily theoretical knowledge. Channels include department-organized study sessions, hospital-led training, and self-directed learning through relevant WeChat official accounts on my phone.”

##### 3.2.1.2 Applicability of innovation (−): discrepancy between theory and clinical practice

Study participants generally believe that the evidence in existing guidelines or consensus documents shows a significant gap compared to the complex realities of the operating room, failing to adequately adapt to the dynamic changes during surgery and the individualized needs of different surgical procedures. P1: “The current pressure injury prevention standards are generally applicable, but when actually implemented, there are certain discrepancies with clinical practice. Some aspects are not detailed enough. For example, patients in the operating room undergoing procedures like liver transplants, nephrectomies, lung surgeries, or orthopedic surgeries such as cervical and lumbar spine surgeries have longer operating times. Patients must remain immobilized throughout the procedure and are completely unable to move.” P4: “The main issue is the significant difference in procedures between the ward and the operating room. For example, in the ward, patients can be repositioned every two hours, but for patients undergoing surgeries lasting more than two hours, the risk of pressure injury is high. In practice, we sometimes find that even when a patient is at high risk, we are unable to reposition them. This differs from—and even conflicts with—the evidence-based prevention knowledge we have.”

#### 3.2.2 Theme 2: external environmental factors

##### 3.2.2.1 Policies and laws (+): strong policy and regulatory support

Policies and regulations at the national and industry levels regarding perioperative patient safety and the prevention and control of pressure injuries have driven the implementation of IAPI prevention efforts, providing institutional support and resource guarantees for the implementation of these measures. All healthcare professionals interviewed in this study agreed that institutional safeguards at the policy and regulatory level have a positive impact on the implementation of IAPI prevention efforts. P7: “Some of the current workflows in our department are carried out in accordance with the relevant guidelines of the Chinese Nursing Association.” P1: “We frequently attend various training sessions. For example, our department has a specialized pressure injury team, and the team members appear to be national-level pressure injury management specialists.”

#### 3.2.3 Theme 3: internal environmental factors

##### 3.2.3.1 Available resources (−): shortages of departmental supplies

The effective implementation of IAPI prevention measures relies on adequate and appropriate prevention tools; sufficient supplies help ensure that these measures are carried out. Respondents in this study generally believe that the department currently has shortcomings in the provision of IAPI prevention-related supplies. There are insufficient quantities of certain protective items, such as positioning cushions and pressure-relieving dressings; in addition, some interviewees noted that the department lacks certain equipment required for evidence translation. P11: “The main issue right now is insufficient quantity. When there are many surgeries or operating rooms are in use, we run out of position cushions. There often aren't enough to go around, and we even have to “fight over them.” When supplies are low, we just have to make do. For example, if I need a turning cushion for a patient in the lateral position but none is available, I have to find something else to substitute—but I can't delay the surgery. So I hope the department can appropriately increase the number of position cushions.” P10: “Often, the tools mentioned in the guidelines or the recommended supplies I've learned about are not available at our hospital. I'm also unsure whether those recommended supplies actually have any practical effect on pressure injury prevention for our patients.”

##### 3.2.3.2 Incentive mechanisms (–): lack of positive incentive mechanisms

The extra effort nurses put into implementing preventive measures, optimizing nursing processes, and participating in quality improvement often fails to receive the recognition and rewards it deserves, and there is a lack of corresponding support for their professional development. Respondents in this study reported that, in their current clinical practice, there is a lack of reasonable positive incentive mechanisms for IAPI prevention efforts. P6: “There doesn't seem to be anything positive to help us do a good job with prevention, because there isn't a reward system. There's no mention of any rewards for preventing a certain number of patients from developing pressure injury.” P12: “pressure injury in patients must be reported; they cannot be concealed. Moreover, there are no rewards or penalties—reporting them does not result in punishment.”

##### 3.2.3.3 Communication (–): poor interdepartmental communication channels

Preventing IAPI requires collaboration among multiple departments; the operating room, surgical department, and anesthesiology department must coordinate and share information in a timely manner. However, study participants generally believe that current interdepartmental communication channels are significantly inadequate, with a lack of fixed, standardized communication mechanisms and information-sharing channels between different departments. P6: “We have almost no direct communication with the wards because, in most cases, we only hand over information to the recovery room during shift changes, and then the recovery room passes it on to the wards. So, we have very little contact with the wards.” P7: “The ward nurses don't really understand our surgeries. For example, when they see redness on a patient's heels, they think there's a problem, but we consider it normal—because if the patient is in the prone position, their heels don't touch the bed, so how could there be pressure injuries?”

##### 3.2.3.4 Shared goal (+): A common desire to reduce the incidence of IAPI

Reducing IAPI is a shared priority for all operating room nurses. The participants in this study believe that, as the direct implementers of IAPI prevention, operating room nurses share a common desire to lower the incidence of IAPI among patients and ensure the safety of perioperative care. P1: “I have always hoped to ensure patients undergo surgery smoothly and recover as soon as possible, while minimizing all types of harm caused by surgery. After all, once pressure injury occur, they can even endanger the patient's life in severe cases.” P10: “Although pressure injury reporting is non-punitive, if a patient under our care develops a pressure injury—which affects their recovery—we also feel a psychological burden.”

##### 3.2.3.5 Accessibility of knowledge and information (+): availability of guidance or training to implement and promote innovation

Study participants generally agreed that their departments have convenient access to guidelines, standards, and implementation instructions related to IAPI prevention. Additionally, specialized training, case discussions, and practical drills are routinely conducted, helping them master key points of the protocols and operational standards, thereby providing the knowledge and capacity needed to implement innovations. P3: “The department leadership has been very supportive. The department generally organizes training sessions on pressure injuries for us, with the goal of ensuring that everyone takes pressure injury prevention seriously.” P5: “The hospital and department conduct regular specialized training on pressure injury prevention and explain the latest requirements from authoritative guidelines. When we encounter operational issues, specialist instructors are always available to provide guidance.”

#### 3.2.4 Theme 4: individual characteristics

##### 3.2.4.1 Capabilities (–): variations in individual capabilities

The standardized implementation of IAPI prevention places high demands on the professional capabilities of operating room nurses. Nurses must not only be proficient in core preventive procedures—such as risk assessment, patient positioning, and pressure-relief interventions—but also possess the ability to handle emergency situations that may arise during surgery. Respondents in this study indicated that there are significant individual differences among department nurses in terms of knowledge, practical skills, and risk assessment capabilities regarding IAPI prevention; some nurses are unable to strictly adhere to clinical guidelines when implementing standardized preventive measures. P3: “Most of my colleagues in the department have a very positive attitude. However, there are inevitably differences in work ability; some are more capable, while others are less so. Some senior nurses really want to do a good job and are already very careful, but sometimes they still feel overwhelmed.” P7: “Some nurses, though well-intentioned and concerned that patients might be cold, take incorrect measures that actually increase the patient's burden. For example, they might place warm items on a patient who is shivering but neglect to monitor and adjust them. Since anesthetized or anxious patients have reduced sensitivity, pressure injury can develop in areas that would not normally be at risk. What could have been avoided ends up increasing the risk because of well-meaning but misguided methods.”

##### 3.2.4.2 Motivation (–): empiricism and complacency

In actual clinical practice, some nurses tend to rely on past work experience and do not place sufficient importance on the latest nursing guidelines, failing to strictly follow the latest protocols when performing procedures related to IAPI prevention. Additionally, the operating room environment is highly demanding and fast-paced; nurses working in such conditions for extended periods are prone to complacency, which to some extent affects the effectiveness of implementing IAPI prevention protocols. P4: “Most of the preventive measures I implement come from my personal work experience, combined with self-reflection and summarization. For example, if I failed to take certain protective measures during a surgery and the patient developed postoperative skin erythema, I would take those measures the next time I encountered a similar procedure. If the patient did not develop skin erythema postoperatively, I would continue to use those effective protective measures.” P3: “Diligent and conscientious nurses will proactively implement existing protocols and related tasks; however, some nurses are more lax or lack the necessary skills, so they do not take the initiative.” P6: “Some nurses lack initiative; they assume that since they have always provided care in this manner without any pressure injury occurring, they can let their guard down. They still fall short in terms of initiative and sense of responsibility.”

##### 3.2.4.3 Leaders (+): leadership support

The attention and support from department and hospital administrators are crucial drivers for the smooth implementation of IAPI prevention efforts, providing the necessary resources to ensure innovative measures are effectively implemented. Respondents in this study indicated that department leaders place high priority on the quality of IAPI prevention, offering clear support in areas such as policy implementation, process optimization, resource allocation, and staff scheduling. These practices have enhanced nurses' enthusiasm and ability to carry out prevention work. P5: “During our small group lectures, professional training sessions, and large-scale ward rounds, our leaders consistently address topics related to intraoperative pressure injuries.” P11: “Our department leadership places significant importance on this issue; they compile the department's pressure injury incidence rate every month.”

##### 3.2.4.4 Needs (+): empathy for the patient's experience

IAPI exacerbates patients' postoperative suffering, not only causing pain but also delaying the physical recovery process. Nurses' empathy toward patients is a key factor driving their proactive efforts in IAPI prevention. According to the respondents in this study, most operating room nurses deeply empathize with patients' discomfort and needs, prioritizing patient comfort and safety in their work. This empathy motivates nurses to implement protective measures more meticulously and proactively mitigate the risk of PI in patients. P3: “Sometimes after my shift, I simulate the patient's surgical position at home to personally experience what it feels like during surgery. This helps me determine whether the positioning I use is comfortable. If I find it comfortable, the patient should also be comfortable during surgery.” P9: “If circumstances permit, we can lie down on the operating table ourselves to simulate the patient's surgical position, personally feeling where the lying position is uncomfortable and which areas bear the most pressure. Having everyone experience this firsthand for a few minutes makes it much more intuitive.”

#### 3.2.5 Theme 5: factors related to the implementation process

##### 3.2.5.1 Adjustment strategies (+): adapting implementation strategies to actual conditions

During the practical application of the program, it is necessary to flexibly adapt to the type of surgery, the patient's specific condition, and the department's workflow, ensuring that all preventive measures are fully aligned with the clinical environment of the operating room. This implementation model, tailored to clinical realities, not only ensures the core quality of IAPI prevention efforts but also effectively enhances the program's clinical feasibility while increasing frontline nurses' acceptance of the program. Respondents in this study indicated that the clinical implementation of innovative IAPI prevention protocols requires flexible adjustments based on actual clinical practice. P4: “The duration of the surgery leads me to adopt different preventive measures depending on the patient's specific condition. Additionally, different surgeries require different patient positions, and we implement targeted pressure injury prevention measures accordingly.” P10: “First, I assess the patient's physical condition, then decide whether additional protection is needed based on the surgical procedure. For example, for underweight or overweight patients, I place thick blankets or soft foam pads on the operating table. I also consider the surgeon's operating speed; for the same gallbladder surgery, some surgeons can complete it in one hour, while others may take three or four hours. I tailor pressure injury prevention measures for patients according to the specific duration of each surgeon's procedure.”

##### 3.2.5.2 Reflection and evaluation (+): strong self-reflection

Feedback from the study's participants indicates that most nurses in the department possess a strong sense of self-reflection. After completing nursing care for each surgery, they proactively review the implementation of IAPI prevention measures, identify shortcomings and oversights in their procedures, and consciously make improvements in subsequent work. P5: “I definitely reflect on what I didn't do well with the previous patient. For example, whether it was due to an uneven mattress, improper use of positioning cushions, or patient-related factors, I reflect on these issues to try to prevent or reduce the occurrence of pressure injury.” P8: “I've actually encountered this situation before. At the time, it was particularly baffling—I'd think to myself, “I've done everything perfectly, so how could the patient still develop pressure injury?” After a patient develops pressure injury, I'm usually notified either by the nurse in charge of pressure injury management or by a supervisor. Following that, I pay extra attention to the patient's recovery from pressure injury and actively reflect on my work to identify areas for improvement.”

## 4 Discussion

This study applied the CFIR 2.0 framework to explore barriers and facilitators of evidence-based IAPI prevention implementation among operating room nurses in a tertiary hospital in Qinghai Province. Guided by the framework's five core domains, we identified 14 key constructs closely related to implementation outcomes, including 8 facilitators and 6 barriers. The following discussion is structured strictly according to the five domains of CFIR 2.0 to maintain alignment with the theoretical framework and enhance systematicity.

### 4.1 Innovation characteristics domain

Two core constructs were identified in the innovation domain: Diversified access to innovation evidence as a facilitator, and Mismatch between guidelines and operating room clinical reality as a barrier.

#### 4.1.1 Facilitator: diversified access to innovation evidence

Multi-channel access to evidence-based information is a fundamental prerequisite for frontline nurses to adopt evidence-based practices. In this study, nurses obtained IAPI prevention knowledge through multiple pathways, including hospital training, departmental learning sessions, online academic resources, and professional official accounts. Wan et al. (33) also noted in a qualitative study that easily accessible, up-to-date guidelines are a positive factor for PI prevention implementation in clinical settings. Diversified information channels ensure that nurses can continuously obtain updated evidence, laying a solid knowledge foundation for the standardized implementation of preventive measures.

#### 4.1.2 Barrier: mismatch between guidelines and operating room clinical reality

Poor applicability of general PI guidelines to the operating room setting was the most prominent barrier at the innovation level. Unlike studies in high-PI-risk settings such as ICU and geriatric wards, where low awareness of guidelines is often the primary barrier (34, 35), nurses in this study did not lack evidence-based knowledge; instead, they found that core recommendations in general guidelines (e.g., 2 h repositioning) were directly incompatible with surgical constraints.

Authoritative institutions worldwide have issued multiple PI prevention guidelines, but most recommendations are developed for ward and ICU settings, emphasizing repositioning and posture adjustment as core preventive measures (36). In the operating room, however, anesthesia-induced immobilization and surgical procedure restrictions make intraoperative repositioning impossible, creating a direct conflict with evidence-based requirements (37). AORN guidelines (10) also note that intraoperative immobilization, hard operating table surfaces, impaired pressure perception, and inability to actively reposition all contribute to IAPI risk, which is consistent with the practical challenges reported by participants in this study. Furthermore, existing evidence has not established individualized, operable prevention protocols tailored to different surgical types and positions (10, 38), forcing nurses to adjust measures based on personal experience in clinical practice.

This finding highlights a specialty-specific implementation gap: the mismatch between guidelines and clinical reality should not be simply attributed to nurses' unfamiliarity with guidelines or poor compliance, but rather to the insufficient operability of evidence-based recommendations in specialized clinical scenarios. Successful evidence-based implementation requires joint consideration of the evidence itself, organizational processes, resource conditions, and frontline user needs (28, 29). Therefore, future IAPI prevention guidelines should move beyond general recommendations and develop specialized, actionable pathways based on surgical position, operation duration, anesthesia method, and available protective materials.

### 4.2 Outer setting domain

In the outer setting domain, policy and regulatory support was identified as a consistent facilitator, with no barriers detected in this domain.

#### 4.2.1 Facilitator: strong policy and regulatory support

National and industry-level policies on perioperative patient safety and pressure injury prevention and control provide institutional support and resource guarantees for the implementation of IAPI prevention measures. In this study, relevant guidelines issued by professional organizations such as the Chinese Nursing Association, together with hospital-level nursing quality control requirements, provided directional guidance for the department to carry out IAPI prevention work, which is consistent with conclusions from domestic perioperative nursing quality improvement research (39). From an implementation science perspective, top-down policy support creates a good external environment for evidence-based nursing practice, which can effectively reduce the negative impact of internal organizational barriers and help evidence-based programs get put into practice.

### 4.3 Inner setting domain

This domain covers organizational factors within the department that shape implementation quality. Two facilitators and three barriers were identified in this domain.

#### 4.3.1 Facilitators

##### 4.3.1.1 Shared goal alignment

Shared goal alignment across the nursing team was a notable internal facilitator. All participants expressed a common desire to reduce IAPI incidence and ensure patient safety. This collective goal orientation, rooted in professional responsibility, provides intrinsic motivation for prevention work. Our research findings are consistent with those of Appiah et al. (40), indicating that even in resource-limited environments, nurses with a strong sense of professional responsibility are more likely to adhere to preventive measures.

##### 4.3.1.2 Accessible knowledge and training support

Convenient access to guidelines, standards, and specialized training is a core internal enabler for standardized implementation. Multiple studies (41) have reported that systematic training is a positive factor for nurses to carry out PI prevention and helps compensate for shortages in staffing and infrastructure. In this study, the department regularly organized specialized training, case discussions, and practical drills, and specialist instructors were available to provide guidance when operational problems arose. This finding is consistent with the core connotation of “access to knowledge and information” in the CFIR framework, indicating that regular specialized training and accessible guideline resources can provide sustained organizational support for nurses to improve their professional competence and implement measures in a standardized manner.

#### 4.3.2 Barriers

##### 4.3.2.1 Insufficient protective resources

Inadequate quantity and incomplete categories of core protective supplies (such as positioning pads and pressure-relief dressings) constitute a direct material barrier to the implementation of standardized preventive measures. This finding is consistent with qualitative studies on PI prevention barriers among ICU nurses in multiple countries including Turkey and Spain (41), further confirming that resource availability in the inner setting is a key determinant of evidence-based nursing implementation. On high-volume surgery days, shortages of dedicated protective supplies often force nurses to use makeshift substitutes, which compromises the quality of prevention.

##### 4.3.2.2 Lack of positive incentive mechanisms

The absence of targeted positive incentive mechanisms is a notable organizational barrier identified in this study. IAPI prevention is a form of invisible labor: when nurses avoid IAPI through adequate assessment, individualized positioning protection, and postoperative follow-up, these additional efforts are often not readily visible; yet when IAPI occurs, nurses may face pressure related to reporting, follow-up, and quality feedback. If the management model only emphasizes accountability after adverse events while lacking recognition and feedback for high-quality preventive behaviors, nurses' motivation for sustained participation will decline (42). This organizational management issue has received relatively little attention in previous operating room IAPI research, but our findings suggest it is a critical lever for improving implementation quality. Quality management for IAPI prevention should therefore focus on both outcome indicators and process indicators, and establish a reasonable positive incentive system to recognize nurses' proactive prevention efforts.

##### 4.3.2.3 Poor interdepartmental communication

Effective IAPI prevention requires seamless collaboration across multiple departments. However, participants in this study generally reported inadequate communication channels between the operating room and inpatient wards. Currently, information is mostly transferred indirectly through the post-anesthesia care unit, and ward nurses often lack understanding of intraoperative positioning constraints and skin conditions, which may lead to misattribution of pressure injury origins and discontinuities in perioperative management. Relevant studies (28, 29) have shown that multidisciplinary collaboration and standardized communication mechanisms are important factors promoting guideline implementation. Therefore, hospital administrators should establish standardized perioperative handover mechanisms for pressure injury information to ensure continuity of care across departments.

### 4.4 Individual characteristics domain

In the field of individual characteristics, two facilitating factors and two hindering factors were identified.

#### 4.4.1 Facilitators

##### 4.4.1.1 Leadership support

Attention and support from department managers are important drivers for the smooth implementation of IAPI prevention. A previous study (35) indicated that managers' attitudes can act as a barrier to PI prevention, and nurses require positive support from institutional managers. Our study confirmed that department leaders' support in policy promotion, material allocation, and training organization effectively reduced organizational resistance to the implementation of IAPI preventive measures. Leaders' emphasis on IAPI prevention quality, such as monthly incidence statistics and regular quality review, also signals the priority of this work and enhances nurses' attention to prevention practices.

##### 4.4.1.2 Nurse empathy and professional responsibility

Nurses' empathy for patients and strong sense of professional responsibility are core intrinsic drivers of proactive prevention behavior. The literature remains inconsistent regarding the impact of caregivers' sense of responsibility on PI prevention. Etafa et al. (43) found that nurses' positive attitudes toward PI prevention were significantly positively correlated with actual preventive behaviors, but external factors such as heavy workload and insufficient resources weakened this motivation. In contrast, Appiah et al. (44) found that even in resource-limited settings, nurses with a strong sense of professional responsibility were more likely to adhere to preventive measures. Our findings support the view that professional responsibility and empathy are positive factors driving preventive behavior.

Notably, role-taking emerged as a specific manifestation of empathy in this study. Some participants reported simulating surgical positions themselves to experience pressure points and optimize positioning comfort, which helped them develop more patient-centered prevention plans. Existing research (45) has noted that when implementing best evidence, nurses need to engage in role-taking and consider the feasibility and comfort of preventive measures from the patient's perspective. However, this facilitator has rarely been mentioned in previous operating room IAPI research, representing a novel finding of this study.

#### 4.4.2 Barriers

##### 4.4.2.1 Variations in individual competence

The standardized implementation of IAPI prevention places high demands on nurses' professional competence. Participants reported significant individual differences among department nurses in terms of IAPI prevention knowledge, practical skills, and risk assessment ability. Some nurses, despite good intentions, adopted incorrect practices (such as improper warming measures) that inadvertently increased PI risk (46). Uneven individual competence leads to inconsistencies in implementation quality across different nurses and different surgical teams.

##### 4.4.2.2 Empiricism and complacency

The present study found that some nurses tend to rely on prior clinical experience rather than strictly adhering to up-to-date evidence-based guidelines in daily practice. Li et al. (47) reported that some nursing staff still hold the view that preventive care can be delivered entirely through experience and intuition, yet the overall quality of their clinical practice is generally suboptimal. This same phenomenon was also observed in the current study. It should be emphasized that the empiricism documented in this study should not be simply construed as nurses' rejection of evidence-based practice. Rather, it reflects the clinical adaptations made by nurses in response to guideline misfit, resource constraints, and time pressure within the complex operating room setting. To a certain extent, tailoring preventive measures based on previous clinical cases and personal experience embodies frontline clinical wisdom. However, when such adjustments lack standardization and evidence-based validation, they may lead to inconsistent implementation of preventive practices and unwarranted variation in nursing quality. Accordingly, future quality improvement initiatives should not merely mandate that nurses “reduce their reliance on experience”. Instead, efforts should focus on translating frontline practical experience into discussable, verifiable, and generalizable specialized operating room practice protocols.

### 4.5 Implementation process domain

Two facilitators were identified in the implementation process domain, and no barriers were found.

#### 4.5.1 Facilitators

##### 4.5.1.1 Flexible strategy adaptation

In the present study, nurses flexibly tailored prevention strategies based on surgical type, operative duration, patient physical status, and surgeon-specific characteristics. Existing evidence (48) demonstrates that by identifying patient risk profiles and accounting for the specific surgical context (e.g., operative duration, procedure type, and surgeon practice patterns), nurses can significantly reduce the incidence of complications and enhance patient satisfaction. This aligns with the conclusions drawn from study participants. This flexibility is reflected in staffing arrangements, intraoperative patient positioning coordination, and the development of individualized rehabilitation plans. Prior research (49) has confirmed that individualized nursing adjustments can significantly decrease the occurrence of postoperative adverse events and reduce hospital length of stay. Collectively, these findings suggest that the flexible adaptation of evidence-based innovations to local clinical contexts may contribute to improving the quality of nursing care.

##### 4.5.1.2 Active self-reflection and evaluation

In contemporary operating room management, reflective practice among nurses has emerged as a pivotal approach to advancing nursing quality. Existing evidence (50) indicates that reflective nursing practice enables nurses to continuously optimize work processes and reduce the incidence of adverse events while delivering continuous, holistic patient care. In the present study, participants reported that most nurses in the department demonstrate strong self-reflective awareness. Following each surgical procedure, they proactively review the implementation of IAPI prevention measures, identify gaps in practice, and implement targeted improvements in subsequent clinical work. A prior study (51) found that nurses with robust self-reflective awareness continuously revise and refine improvement measures across all phases of perioperative care through a postoperative review mechanism. Clinical practice data show (52) that maintaining reflective diaries and summarizing practical experience not only elevates patient satisfaction with nursing services but also enhances nurses' professional fulfillment.

Based on participant narratives, we propose an emergent conceptual model derived from qualitative analysis: the core issue is the mismatch between general PI guidelines and the specialized operating room context, which further triggers a series of organizational and individual adaptive responses such as resource constraints, lack of incentives, and experience-based practice. Facilitators including policy support, leadership engagement, nurse empathy, and flexible implementation can partially mitigate these barriers, but cannot fully compensate for the core problem of guideline-specialty misfit.

**Figure 2:**
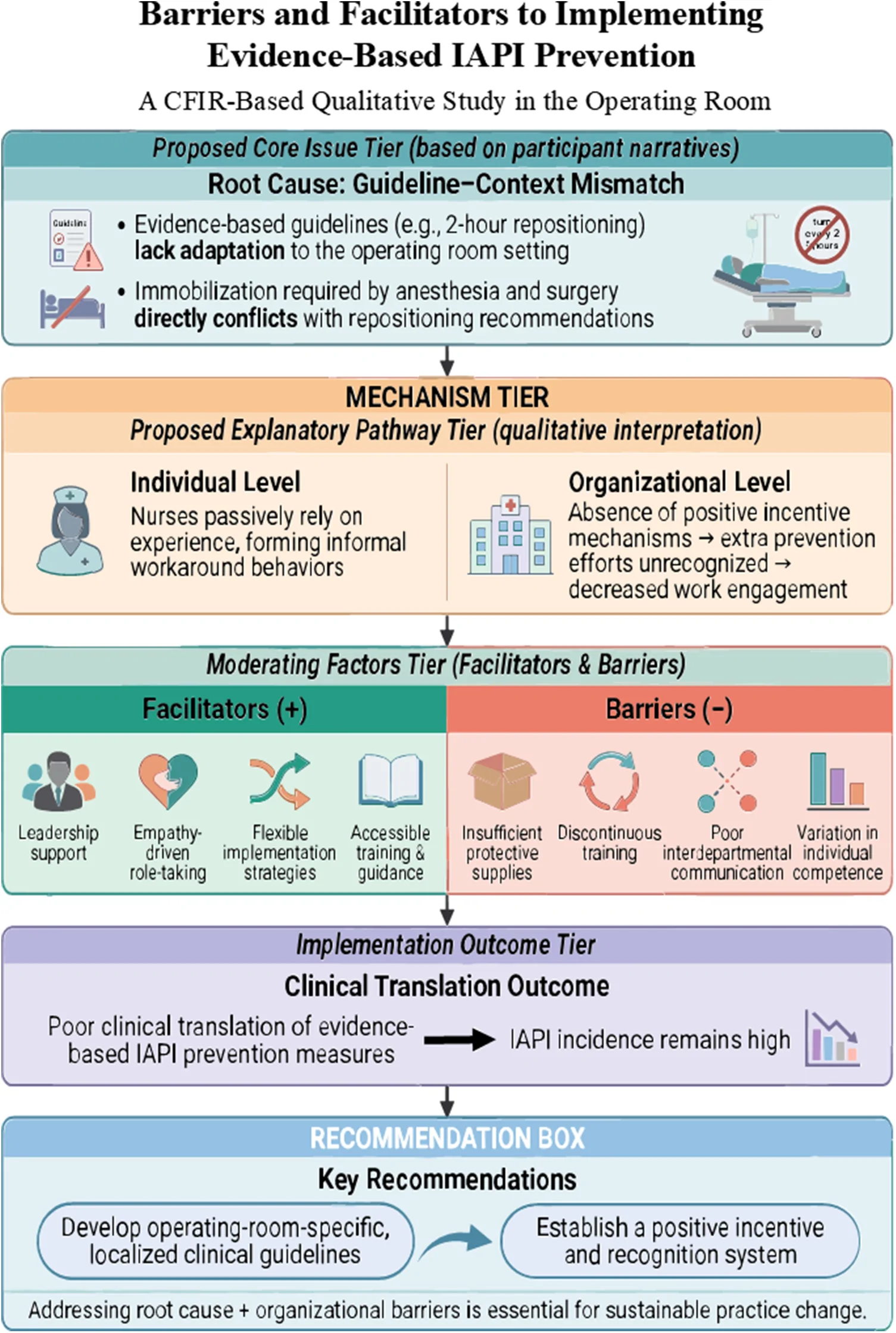
Emergent conceptual model of evidence-based IAPI prevention implementation barriers: A qualitative interpretation based on participant narratives.

It should be explicitly clarified that this model is a conceptual interpretation emerging from qualitative analysis, not a proven causal chain. The pathway described is based on the thematic interpretation of participant narratives and requires further verification through quantitative and interventional research in the future (Figure 2).

## 5 Clinical implications and recommendations

This study represents the first attempt to describe the barriers and facilitators to evidence-based IAPI prevention practices among operating room nurses using the CFIR 2.0 framework. The findings provide a theoretical basis for institutional administrators and the public healthcare sector to implement measures and develop plans aimed at improving nurses' behaviors in clinical settings, thereby reducing the incidence of PI.

Innovative evidence is a key facilitator of implementation, and robust innovative evidence can bridge the gap between evidence-based practice and research. In this study, our interviews revealed that while current international guidelines clearly outline standardized prevention and control strategies, the evidence underlying these guidelines may be difficult to implement in underdeveloped regions—a finding consistent with previous literature. This may be attributed to the fact that certain high-tech pressure-relief devices or dressings recommended by international guidelines may be prohibitively expensive or difficult to obtain in China, particularly in primary care facilities. At the same time, nursing staff shortages make it difficult to implement the time-consuming and labor-intensive preventive measures recommended in the guidelines. Nanna et al. (53) noted in their study that developing localized clinical guidelines helps overworked healthcare workers in resource-poor hospitals improve the quality of daily nursing care. Therefore, future research should focus on implementation science, conducting specialized studies on the barriers and facilitators in localized practice to provide a basis for developing more effective dissemination strategies. Furthermore, health administrative departments and hospital institutions should take into account the constraints of resource-limited settings and collaborate with frontline healthcare workers and scientific experts to develop guidelines, ensuring both safety and feasibility.

In nursing management, positive incentive mechanisms play a crucial role. As direct participants in pressure injury (PI) prevention, nurses can only achieve effective preventive outcomes through proactive, active, and effective implementation of preventive care measures. This study found that the absence of positive incentive mechanisms prevents nurses’ additional efforts in the Interventional Pressure Injury Prevention (IAPI) program from receiving recognition and feedback, leading to reduced job satisfaction and diminished work engagement—a finding consistent with that of Lotfi et al. (54). Concurrently, Chang et al. (55) noted in their study that implementing incentive-based management strategies in PI prevention can reduce the risk of PI occurrence and enhance nursing staff's ability to prevent pressure injuries. This indicates that a well-designed and reasonable positive incentive mechanism can effectively mobilize nurses' enthusiasm for participating in the prevention of intraoperative pressure injuries, improve the implementation of protective behaviors, and consequently enhance the quality of PI prevention. It is recommended that a targeted positive incentive system be established and improved in operating room nursing management. Timely recognition, feedback, and appropriate incentives should be provided for nurses’ extra efforts and proactive behaviors in pressure injury prevention. This will enhance nurses' job satisfaction and initiative, promote the sustained implementation of standardized preventive measures, and reduce the incidence of intraoperative pressure injuries.

## 6 Conclusion

Using the CFIR 2.0 framework, this study systematically identified the facilitating and hindering factors influencing the implementation of evidence-based IAPI prevention measures by operating room nurses. Major barriers included insufficient alignment between evidence-based guidelines and clinical practice, shortages in departmental supplies, lack of positive incentive mechanisms, poor interdepartmental communication, variations in individual nurse competencies, as well as a tendency toward empiricism and complacency. We found that the clinical translation of evidence-based IAPI prevention measures in the operating room is the result of the combined effects of multidimensional factors; therefore, improving the implementation of these measures requires overcoming these challenges. In the future, based on the core influencing factors identified in this study, a system for the implementation of evidence-based IAPI prevention practices tailored to the specialized operating room setting can be established to effectively reduce the incidence of IAPI in surgical patients and improve perioperative nursing quality.

### 6.1 Limitations

This study has certain limitations: First, the study only selected operating room nurses from a single tertiary hospital in Qinghai as the research subjects. The geographical scope and level of the healthcare institution were relatively limited, which restricts the generalizability of the findings; Additionally, due to the potential subjectivity of interviews and the possibility that respondents may withhold sensitive information, the findings of this qualitative study cannot be generalized to a broader population. Future quantitative studies are needed to understand the overall healthcare status of this population and comprehensively investigate its influencing factors. Second, all interviews were conducted by researchers from the same institution. Although no direct supervisory relationship existed between the interviewer and participants, the potential for social desirability bias cannot be fully excluded, which may have led participants to understate criticisms of institutional management or departmental leadership.

## Data Availability

The original contributions presented in the study are included in the article/Supplementary Material, further inquiries can be directed to the corresponding author/s.
